# CD45RA^-^Foxp3^high^ but not CD45RA^+^Foxp3^low^ suppressive T regulatory cells increased in the peripheral circulation of patients with head and neck squamous cell carcinoma and correlated with tumor progression

**DOI:** 10.1186/1756-9966-33-35

**Published:** 2014-04-25

**Authors:** Wei Sun, Wei-Jin Li, Chang-You Wu, Hua Zhong, Wei-Ping Wen

**Affiliations:** 1 Department of Otorhinolaryngology Head and Neck Surgery, the First Affiliated Hospital of Sun Yat-sen University, 2nd Zhongshan Road 58#, Guangzhou 510080, Guangdong, P.R. China; 2 Institute of Otorhinolaryngology Head and Neck Surgery, Sun Yat-sen University, 2nd Zhongshan Road 58#, Guangzhou 510080, Guangdong, P.R. China; 3 Institute of Immunology, Zhongshan School of Medicine, Sun Yat-sen University, 2nd Zhongshan Road 74#, Guangzhou 510080, Guangdong, P.R. China

**Keywords:** CD45RA, Foxp3, T regulatory cells, Head and neck squamous cell carcinoma

## Abstract

**Background:**

T regulatory cells (Tregs) contribute to the progression of head and neck squamous cell carcinoma (HNSCC) by suppressing antitumor immunity. However, little is known regarding the functional heterogeneity of Tregs in HNSCC patients.

**Methods:**

Using multicolor flow cytometry, the frequency of three Treg subsets, separated on the basis of CD45RA and Foxp3, from the peripheral circulation of newly-presenting HNSCC patients (19 oral cavity squamous cell carcinoma, 20 hypopharyngeal squamous cell carcinoma, 18 nasopharyngeal squamous cell carcinoma, 19 oropharyngeal squamous cell carcinoma, and 36 laryngeal squamous cell carcinoma) were assessed with regard to 31 healthy donors and clinicopathological features. Moreover, the functional capacity of each Treg subsets was evaluated based on CD45RA and CD25 expression.

**Results:**

The frequency of Tregs in the peripheral circulation of HNSCC patients as a whole cohort was higher than in healthy donors (P < 0.0001). However, the frequency of Tregs was similar between patients with oral cavity squamous cell carcinoma and healthy donors (P = 0.269). Further dividing Tregs into three subsets based on Foxp3 and CD45RA expression revealed that the frequency of CD45RA^-^Foxp3^high^ Tregs and CD45RA^-^Foxp3^low^CD4^+^ T cells in patients with HNSCC developing from different subsites was higher than in healthy donors (P < 0.0001, P < 0.0001), whereas the frequency of CD45RA^+^Foxp3^low^ Tregs was lower than in healthy donors (P < 0.0001). Functionally study revealed that CD45RA^-^CD25^+++^ Tregs significantly inhibit the proliferation of CD4^+^CD25^-^ T cells (P < 0.001) and secrete lower levels of cytokines (P < 0.01) compared with CD45RA^-^CD25^++^CD4^+^ T cells. Importantly, the frequency of CD45RA^-^Foxp3^high^ Tregs positively correlate with tumor stage (P < 0.0001) and nodal status (P < 0.0001).

**Conclusions:**

CD45RA^-^Foxp3^high^ Tregs increase in the peripheral circulation of HNSCC patients, and correlate with tumor stage and nodal status; suggesting a role in tumor progression which may be manipulated by future immunotherapy.

## Background

Globally, head and neck cancer is the sixth most common type of cancer [1]. Approximately 90% of head and neck cancer cases arise from organs lined by squamous epithelium [2]. Despite new treatment modalities (including surgical and adjuvant chemoradiotherapy) and their success in terms of overall quality of life, survival rates for this disease have not improved in the past 30 years [3].

It is widely recognized that the progression of head and neck squamous cell carcinoma (HNSCC) is attributed to the peripheral immune tolerance to tumors [4]. Foxp3^+^CD25^+^CD4^+^ T regulatory cells (Tregs), with immunosuppressive activity against tumor-specific T cell responses, are one of the crucial players for immune tolerance [5,6].

To date, Tregs have been shown to be elevated in a number of different cancers [7-13], including HNSCC where it has been reported that Tregs increase in the peripheral circulation when compared with healthy donors. However, Tregs are not functionally homogeneous [14]. For example, Zhou et al. [15] showed that CD4^+^Foxp3^-^ T cells could transiently express lower levels of Foxp3 and leads to the generation of pathogenic memory T cells. Allan et al. [16] postulated that activated CD4^+^ T cells, but without regulatory activity, could express Foxp3. Hence, identification of distinct Treg subsets and their functional abilities might be more intriguing in antitumor immunity field.

Recently, Sakaguchi’s group demonstrated that human Tregs can be dissected into three functionally distinct subsets on the basis of CD45RA, Foxp3 and CD25 expression: CD45RA^+^Foxp3^low^ Tregs (resting Tregs), which are CD25^++^, CD45RA^-^Foxp3^high^ Tregs (activated Tregs), which are CD25^+++^, and CD45RA^-^Foxp3^low^CD4^+^ T cells (cytokine-secreting non-suppressive T cells), which are CD25^++^[14]. Based on this classification of human Tregs, subsequent studies showed that the frequency and function of these Treg subsets vary in different disease models, including systemic lupus erythematosus, sarcoidosis, and aplastic anemia [14,17,18]. However, the characterizations of these functionally distinct Treg subsets in HNSCC are unknown.

When assessing the Treg subsets it is important not only to examine their characteristics in HNSCC patients as a whole cohort, but also to investigate their variations in patients with HNSCC developing from different anatomic subsites, as the various subsites of HNSCC are known to have different etiology and survival rates. To our knowledge, this is the first study to use the CD45RA, Foxp3, and CD25 markers to study both the frequency and function of three distinct Treg subsets in the peripheral circulation of newly-presenting HNSCC patients in relation to tumor subsites, tumor stage and nodal status.

## Materials and methods

### Patients and healthy donors

From September 2012 to February 2014, 112 HNSCC patients were enrolled in the present study [19 oral cavity squamous cell carcinoma (OCSCC), 20 hypopharyngeal squamous cell carcinoma (HPSCC), 18 nasopharyngeal squamous cell carcinoma (NPSCC), 19 oropharyngeal squamous cell carcinoma (OPSCC), and 36 laryngeal squamous cell carcinoma (LSCC)]. Patients were diagnosed at the Department of Otorhinolaryngology, the First Affiliated Hospital of Sun Yat-sen University without any previous oncological treatment. Healthy age-matched donors (29 males and 2 female with a mean age of 45 years; range: 38–81) were enrolled as controls. The main clinical and pathologic characteristics of the patients are presented in Table 1. Clinical staging and the anatomic subsites of the tumors were assessed according to the 6th edition of the Union Internationale Contre Cancer (UICC 2008) tumor-node-metastasis classification of malignant tumors.

**Table 1:** Clinicopathological features of 112 HNSCC patients who donated peripheral blood for this study

**Characteristics**	**Number**
**Age (years) mean (range)**	47 (37–83)
**Gender**	
Male	108
Female	4
Total	112
**Tumor site**	
Oral cavity	19
Hypopharynx	20
Nasopharynx	18
Oropharynx	19
Larynx	36
**Tumor stage**	
T_1–2_	46
T_3–4_	66
**Nodal status**	
N_0_	70
N^+^	42
**M stage**	
M_0_	112
M_1_	0

*HNSCC*, Head and neck squamous cell carcinoma.

### Ethics statements

The study protocol (No. 2012–349) was approved by the ethic Committee of The First Affiliated Hospital of Sun Yat-sen University, and was used for research purposes only. Patient and healthy donor (HD) informed consent was obtained before enrollment.

### Collection of peripheral blood

Peripheral blood lymphocytes (PBLs) were isolated from peripheral venous blood as previously described [19]. Isolated cells were immediately re-suspended in 100 μl flow cytometry staining buffer (eBioscience, San Diego, CA, USA) for surface and intracellular staining.

### Antibodies and reagents

Freshly obtained human PBLs were stained with the following anti-human monoclonal antibodies: anti-CD3-eFluor 605NC (0.25 μg/100 μl), anti-CD4-FITC (1.0 μg/100 μl), anti-CD25-APC (0.125 μg/100 μl), and anti-CD45RA-eFluor 450 (0.5 μg/100 μl) for surface staining. Anti-Foxp3-PE (0.25 μg/100 μl), anti-tumor necrosis factor-alpha (TNF-α)-Alexa Fluor 700 (0.25 μg/100 μl), anti-interleukin-2 (IL-2)-PE-Cy7 (0.125 μg/100 μl), anti-interferon-gamma (IFN-γ)-APC-eFluor780 (0.25 μg/100 μl), and anti-hinterleukin-17 (IL-17)-PerCP-Cy5.5 (0.125 μg/100 μl) for intracellular staining. Soluble anti-CD3 (OKT3, 0.5 μg/ml) and anti-CD28 (CD28.2, 2 μg/ml) mAb were used for in vitro activation of T cells. All antibodies and isotype controls were purchased from eBioscience (San Diego, CA, USA).

### Multicolor flow cytometry

Multicolor flow cytometry was done using a Ten-Color (3 laser: 488 nm blue, 638 nm red, and 405 nm violet) Gallios Flow Cytometer (Beckman Coulter, Hercules, CA, USA) equipped with Gallios Software v1.0. The acquisition and analysis gates for PBLs (5 × 10^4^) were determined by characteristic forward and side-scatter properties of lymphocytes. Furthermore, analysis gates were restricted to the CD3^+^CD4^+^ T-cell subsets. CD45RA^+^Foxp3^low^ Tregs (I), CD45RA^-^Foxp3^high^ Tregs (II), and Foxp3^low^CD45RA^-^ T cells (III) were determined as previously described [14]. Cells expressing surface and intracellular markers were acquired and analyzed on a logarithmic scale from FL1 to FL9.

### Surface and intracellular staining

To determine the frequency of three distinct Treg subsets, both cell surface and intracellular staining was performed. Briefly, mAbs against surface markers CD3, CD4, CD25, and CD45RA were added to the cell suspension (1 × 10^7^ cells/100 μl) and incubated on ice for 30 minutes in the dark. After washing twice, cells were fixed and permeabilized on ice with fixation/permeabilization buffer (eBioscience, San Diego, CA, USA) for 1 hour in the dark. Cells were then washed twice and incubated with intracellular mAbs for 1 hour at room temperature in the dark. After intracellular staining, cells were washed twice and examined by multicolor flow cytometry. Appropriate isotype Ab controls were included for each sample.

### Cell culture

RPMI 1640 medium supplemented with 10% fetal bovine serum, 100 IU/ml penicillin, and 100 mg/ml streptomycin (Sigma, St. Louis, MO) was used for T cell culture.

### In vitro suppression assay of three distinct Treg subsets

Stained cells (mAbs against CD3, CD4, CD25, and CD45RA) at a concentration of 5 × 10^7^ cells/100 μl were sorted using a FACS cell sorter (BD Influx, BD Biosciences). Three Treg subsets were prepared as live cells as previously described [14]; i.e., Foxp3^low^CD45RA^+^ (I), Foxp3^high^CD45RA^-^ (II), and Foxp3^low^CD45RA^-^ cells (III) were prepared by sorting as CD25^++^CD45RA^+^, CD25^+++^CD45RA^-^, and CD25^++^CD45RA^-^CD4^+^ T cells, respectively. For HNSCC patients, Additional file 1: Figure S1 demonstrates that the degree of CD25 expression in CD45RA^+^CD25^++^ Tregs, CD45RA^-^CD25^+++^ Tregs, and CD45RA^-^CD25^++^CD4^+^ T cells are proportional to Foxp3 expression in CD45RA^+^Foxp3^low^ Tregs, CD45RA^-^Foxp3^high^ Tregs, and CD45RA^-^Foxp3^low^ CD4^+^ T cells, respectively.

After sorting, 1 × 10^4^ responder cells (CD25^-^CD45RA^+^CD4^+^ T cells) were labeled with 1 μM carboxyfluorescein diacetate succinimidyl ester (CFSE) (eBioscience, San Diego, CA, USA) and co-cultured with unlabeled CD25^++^CD45RA^+^, CD25^+++^CD45RA^-^, or CD25^++^CD45RA^-^ CD4^+^ T cells and assessed for their suppressive activities. Soluble anti-CD28 (2 μg/ml) and plate-bound anti-CD3 (0.5 μg/ml) was used to activate T cells in 96-well round-bottom plates, and cells harvested and analyzed by flow cytometry after 86 h of co-culture. All CFSE data were analyzed using the ModFit software provided by Verity Software House (Topsham, USA). The percentages of suppression were determined based on the proliferation index (PI) of responder cells alone (100% proliferation, 0% suppression) compared with the PI of responders co-cultured (1:1 ratio) with each Treg subset.

### Secretory functions of three distinct Treg subsets

To examine secretory function, sorted CD25^++^CD45RA^+^, CD25^+++^CD45RA^-^, or CD25^++^CD45RA^-^ CD4^+^ T cells were stimulated with a cocktail of phorbol 12-myristate 13-acetate (PMA), ionomycin, and Golgi stop (brefeldin A and monensin) (eBioscience, San Diego, CA, USA) for 5 h. Then, intracytoplasmic expression of IL-2, IL-17, TNF-α, and IFN-γ were assessed using intracellular staining.

### Statistical analysis

Statistical analysis was performed with the SPSS software (SPSS Standard version 13.0, IBM, Chicago, IL, USA). The Mann–Whitney U-test or Kruskal–Wallis test was used for analyzing differences between data sets without normal distribution. Differences between independent data sets, with normal distribution, were analyzed using the Student's t-test.

## Results

### Prevalence of three distinct Treg subsets in the peripheral circulation of 112 HNSCC patients

**Figure 1:**
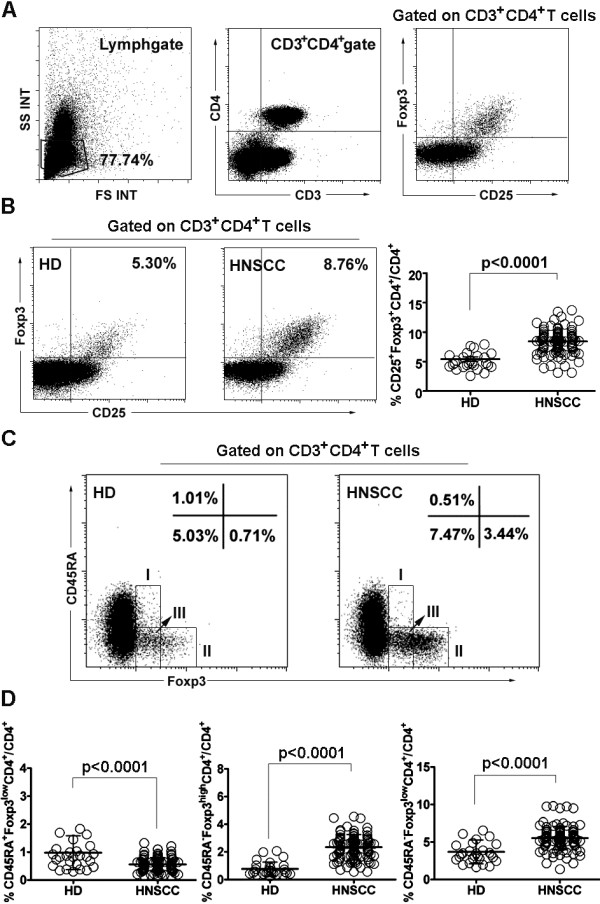
**Percentage of Treg subsets in 112 HNSCC patients. (A)** Gating strategy used is illustrated. **(B)** Flow dot plots of Foxp3^+^CD25^+^ Tregs for one representative HD (left) and HNSCC patient (middle). Percentage (means ± SD) of Foxp3^+^CD25^+^ Tregs in HNSCC patients or HD (right). **(C)** Flow dot plots of each Treg subset (I: CD45RA^+^Foxp3^low^ Tregs; II: CD45RA^-^Foxp3^high^ Tregs; III: CD45RA^-^Foxp3^low^CD4^+^ T cells) for one representative HD (left) and HNSCC patient (right). **(D)** Percentage (means ± SD) of each Treg subset in HNSCC patients or HD. HNSCC: head and neck squamous cell carcinoma. HD: healthy donors. Statistical comparisons were performed using the Mann–Whitney U-test.

Figure 1A illustrates the gating strategy used to identify the frequency of CD25^+^Foxp3^+^ Tregs in the total CD3^+^CD4^+^ T cells. The frequency of these Tregs in the peripheral circulation of HNSCC patients as a whole cohort was higher than in HD (8.12 ± 2.34% vs. 5.44 ± 1.92%, P < 0.0001) (Figure 1B), consistent with previous findings [10]. The frequency of three Treg subsets was then evaluated based on CD45RA and Foxp3 expression. The novelty of this study was that the frequency of CD45RA^-^Foxp3^high^ Tregs (2.23 ± 0.98% vs. 0.77 ± 0.49%, P < 0.0001) and CD45RA^-^Foxp3^low^CD4^+^ T cells (5.36 ± 1.63% vs. 3.70 ± 1.58%, P < 0.0001) in HNSCC patients was higher than in HD, whereas the frequency of CD45RA^+^Foxp3^low^ Tregs in HNSCC patients was lower than in HD (0.53 ± 0.24% vs. 0.98 ± 0.61%, P < 0.0001) (Figure 1C, D).

### Suppressive and secretory function of three distinct Treg subsets

**Figure 2:**
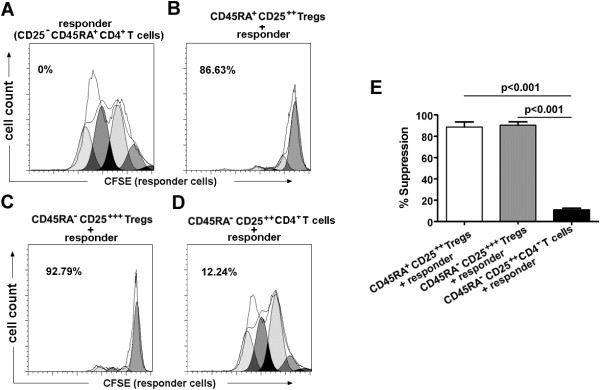
**Percentage of suppression by each Treg subset on the proliferation of responder T cells. (A-D)** CFSE dilution by 1 × 10^4^ labeled CD4^+^CD25^-^CD45RA^+^ T cells (responder T cells) assessed after 86 hr of TCR-stimulated co-culture with indicated Treg subset at a 1 to 1 ratio. Flow plots for one representative HNSCC patient. Percentage of suppression is indicated. In each histogram, the lines indicate the parent population (parent line) and the generation population (generation line) 1, 2, 3… from right to left. **(E)** The histogram represents the mean percentages of suppression ± SD (n = 12). HNSCC: head and neck squamous cell carcinoma. Statistical comparisons were performed using the Student’s t-test.

The suppressive activity of each Treg subset from 12 randomly selected HNSCC patients was assessed by their ability to suppress the proliferation of autologous T cell populations (CD25^-^CD45RA^+^CD4^+^). When each Treg subset isolated from HNSCC patients was co-cultured (1:1 ratio) with CD25^-^CD45RA^+^CD4^+^ responder cells, both CD45RA^-^CD25^+++^ and CD45RA^+^CD25^++^ Tregs consistently induced a greater percentage of suppression compared with CD45RA^-^CD25^++^CD4^+^ T cells (90.34 ± 3.22% vs. 10.81 ± 1.64%, P < 0.001; 88.60 ± 4.86% vs. 10.81 ± 1.64%, P < 0.001, respectively) (Figure 2A-E). However, each Treg subset didn’t inhibit the cytokine production by responder cells (P > 0.05) (Additional file 2: Figure S2), which is in parallel with previous studies [20,21].

**Figure 3:**
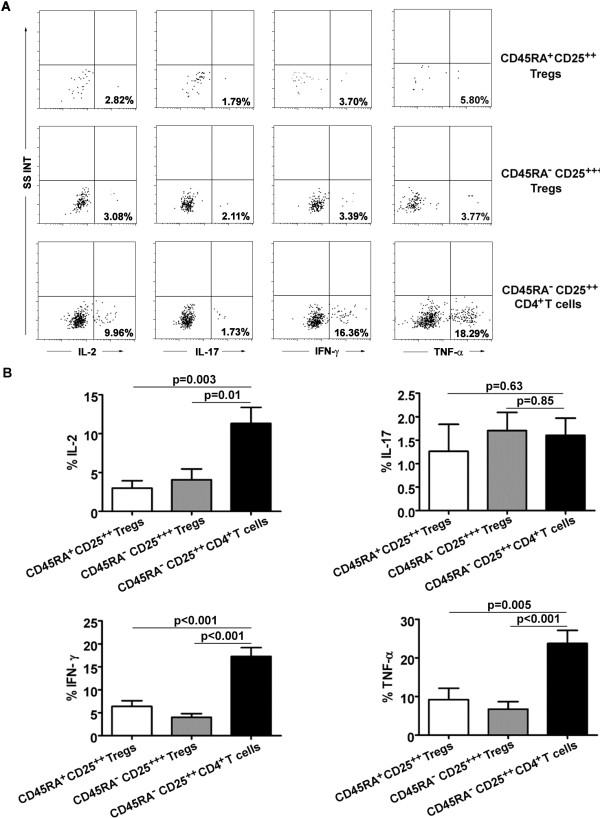
**Cytokine production of each Treg subset. (A)** Production of IL-17, IL-2, IFN-γ, and TNF-α by each Treg subset after stimulation with PMA + ionomycin, and percentage of cytokine-secreting cells in each Treg subset is shown. Data are representative of 9 separate experiments. **(B)** The histogram represents the cytokine expression profiles of each Treg subset. Statistical comparisons were performed using the Student’s t-test.

Moreover, functional cytokine patterns in three Treg subsets from 9 HNSCC patients were also studied after ex vivo stimulation. Our results showed that CD45RA^-^CD25^++^CD4^+^ T cells secreted significantly higher amount of IL-2 (P = 0.004, P = 0.01), IFN-γ (P < 0.001, P < 0.001) and TNF-α (P < 0.001, P = 0.005) than did CD45RA^-^CD25^++^ or CD45RA^+^CD25^++^ Tregs, whereas IL-17 production remained the same (P > 0.05) (Figure 3A, B).

### Prevalence of three distinct Treg subsets in HNSCC patient subgroups

Dividing the HNSCC patient cohort by tumor subsite demonstrated that the frequency of Tregs in patients with OPSCC (8.93 ± 1.49%, P < 0.0001), LSCC (8.09 ± 1.66%, P < 0.0001), HPSCC (9.68 ± 1.94%, P < 0.0001), and NPSCC (8.58 ± 2.62%, P < 0.0001) was higher than in HD (5.44 ± 1.92%). However, the frequency of Tregs was similar between OCSCC patients and HD (5.70 ± 1.56% vs. 5.44 ± 1.92%, P = 0.269).

The frequency of CD45RA^-^Foxp3^high^ Tregs in patients with OCSCC (1.06 ± 0.36%, P = 0.006), OPSCC (2.54 ± 0.42%, P < 0.0001), LSCC (2.36 ± 0.92%, P < 0.0001), HPSCC (2.51 ± 0.76%, P < 0.0001), and NPSCC (2.69 ± 1.12%, P < 0.0001) was higher than in HD (0.77 ± 0.49%), whereas the frequency of CD45RA^+^Foxp3^low^ Tregs in patients with OCSCC (0.39 ± 0.17%, P < 0.0001), OPSCC (0.52 ± 0.16%, P = 0.002), LSCC (0.62 ± 0.28%, P = 0.008), HPSCC (0.58 ± 0.24%, P = 0.003), and NPSCC (0.55 ± 0.21%, P = 0.002) was lower than in HD (0.98 ± 0.61%).

**Figure 4:**
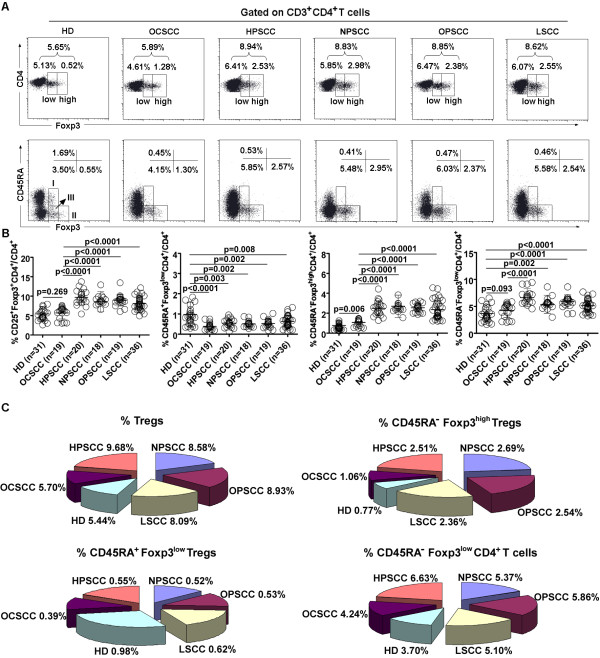
**Percentage of Treg subsets in HNSCC patient subgroups. (A)** Flow dot plots of Tregs (Foxp3^low^ and Foxp3^high^ Tregs) (top) and each Treg subset (I: CD45RA^+^Foxp3^low^ Tregs; II: CD45RA^-^Foxp3^high^ Tregs; III: CD45RA^-^Foxp3^low^CD4^+^ T cells) (bottom) for one representative HD and patients with HPSCC, NPSCC, OPSCC, and LSCC. **(B)** Percentage (means ± SD) of Tregs and each Treg subset in HNSCC patient subgroups or HD. **(C)** Different proportions (means) of each Treg subset in HNSCC patient subgroups are presented. HD: healthy donors. OCSCC: oral squamous cell carcinoma. HPSCC: hypopharyngeal squamous cell carcinoma. NPSCC: nasopharyngeal squamous cell carcinoma. OPSCC: oropharyngeal squamous cell carcinoma. LSCC: laryngeal squanmous cell carcinoma. Statistical comparisons were performed using the Kruskal–Wallis test.

The frequency of CD45RA^-^Foxp3^low^CD4^+^ T cells in patients with OPSCC (5.86 ± 1.26%, P < 0.0001), LSCC (5.10 ± 1.14%, P < 0.0001), HPSCC (6.63 ± 1.67%, P < 0.0001), and NPSCC (5.37 ± 1.66%, P = 0.002) were higher than in HD (3.70 ± 1.58%). However, the frequency of CD45RA^-^Foxp3^low^CD4^+^ T cells was similar between OCSCC patients (4.24 ± 1.31%) and HD (3.70 ± 1.58%) (P = 0.093) (Figure 4A-C).

### Relationship between three Treg subsets and tumor sites

The frequency of CD45RA^-^Foxp3^high^ Tregs in patients with OPSCC (2.54 ± 0.42%, P < 0.0001), LSCC (2.36 ± 0.92%, P < 0.0001), HPSCC (2.51 ± 0.76%, P < 0.0001), and NPSCC (2.69 ± 1.12%, P < 0.0001) was higher than in OCSCC patients (1.06 ± 0.36%). There was no significant difference in the frequency of CD45RA^-^Foxp3^high^ Tregs between patients with OPSCC, LSCC, HPSCC, and NPSCC (P > 0.05). Moreover, there was no significant difference in the frequency of CD45RA^+^Foxp3^low^ Tregs between patients with OCSCC, OPSCC, LSCC, HPSCC, and NPSCC (P > 0.05). The frequency of CD45RA^-^Foxp3^low^CD4^+^ T cells in HPSCC patients was higher than in OCSCC patients (6.63 ± 1.67% vs. 4.24 ± 1.31%, P < 0.0001) (Figure 4B).

### Relationship between three Treg subsets and tumor progression

The frequency of CD45RA^-^Foxp3^high^ Tregs in patients with T_3–4_ or N^+^ was higher than in patients with T_1–2_ or N_0_, respectively (T_3–4_ vs. T_1–2_: 2.81 ± 0.89% vs. 1.83 ± 0.82%, P < 0.0001; N^+^ vs. N_0_: 2.92 ± 1.03% vs. 1.81 ± 0.65%, P < 0.0001).

The frequency of CD45RA^+^Foxp3^low^ Tregs did not differ between patients with T_3–4_ and T_1–2_ (0.52 ± 0.18% vs. 0.54 ± 0.28%, P = 0.834) or with N^+^ and N_0_ (0.50 ± 0.17% vs. 0.55 ± 0.17%, P = 0.556). The frequency of CD45RA^-^Foxp3^low^CD4^+^ T cells in patients with T_3–4_ or N^+^ was higher than in patients with T_1–2_ or N_0_, respectively (T_3–4_ vs. T_1–2_: 6.26 ± 1.39% vs. 4.73 ± 1.49%, P < 0.0001; N^+^ vs. N_0_: 6.07 ± 1.81% vs. 4.93 ± 1.36%, P < 0.0001) (Table 2).

**Table 2:** Relationship between Treg subsets and tumor progression

	**CD45RA**^**-**^**Foxp3**^**high**^	**P**	**CD45RA**^**+ **^**Foxp3**^**low**^	**P**	**CD45RA**^**-**^**Foxp3**^**low**^	**P**
	**Tregs (%)**		**Tregs (%)**		**CD4**^**+ **^**T cells (%)**	
**T**_**1–2**_	1.83 ± 0.82		0.54 ± 0.28		4.73 ± 1.49	
**T**_**3–4**_	2.81 ± 0.89	<0.0001	0.52 ± 0.18	0.834	6.26 ± 1.39	<0.0001
**N**_**0**_	1.81 ± 0.65		0.55 ± 0.17		4.93 ± 1.36	
**N**^**+**^	2.92 ± 1.03	<0.0001	0.50 ± 0.17	0.556	6.07 ± 1.81	<0.0001

Statistical comparisons were performed using the Mann–Whitney U-test.

## Discussion

Tregs have been suggested to contribute to HNSCC progression by suppressing antitumor immunity [4]. Although Tregs in the peripheral circulation of HNSCC patients have been investigated previously, most of these studies were focused on the frequency and suppressive function of CD25^+^ Tregs or CD25^high^ Tregs [10,22-24], and the functional heterogeneity of Tregs was not fully investigated. To expand the understanding of functionally distinct Treg subsets in HNSCC, we recruited a cohort of 112 newly-presenting HNSCC patients that had not received any previous treatment for cancer. The use of the CD45, Foxp3, and CD25 markers has allowed both the frequency and the function of three distinct Treg subsets in the circulation of HNSCC patients with tumors of varying stage and nodal status to be determined.

There is evidence that Tregs are negative prognostic factors for patients with types of human malignancies [7,8,25]. In contrast to these results, however, previous studies of Tregs in HNSCC showed different conclusions. For example, Pretscher et al. [26] showed that higher levels of Tregs do not show any significant influence on outcome of oro- and hypopharyngeal carcinoma patients, and other HNSCC studies even showed that expansion of Tregs is significant prognostic factor related to better locoregional control and overall survival [27,28]. This apparent confusion regarding the role of Tregs in prognosis of cancer patients might be explained by the functional heterogeneity of Tregs or the nature of tumor type, or some combination of the two.

Hence, to understand the heterogeneous role of Tregs, Tregs in the peripheral circulation of 112 HNSCC patients were dissected into three functionally distinct subsets based on the expression of CD45RA, Foxp3, and CD25, and our results showed that although the frequency of Tregs in HNSCC patients was higher than in healthy age-matched donors, which is in agreement with previous studies [10,22], both the frequency and function of these three Treg subsets varied in HNSCC patients; i.e., the frequency of CD45RA^-^Foxp3^high^ suppressive Tregs in HNSCC patients was higher than in healthy donors, whereas the frequency of CD45RA^+^Foxp3^low^ Tregs was lower, suggesting that CD45RA^+^Foxp3^low^ Tregs may be swiftly converted into CD45RA^-^Foxp3^high^ Tregs immediately after migrating from the thymus or having been peripherally generated [14]. Although we are not aware of this phenomenon in human malignancies, the conversion of CD45RA^+^Foxp3^low^ Tregs to CD45RA^-^Foxp3^high^ Tregs has been found in other pathological conditions, such as sarcoidosis [14].

Sakaguchis’s group defined CD45RA^-^Foxp3^low^CD4^+^ T cells as cytokine-secreting non-Tregs for their ability to secrete several cytokines (IL-2, IL-17, and IFN-γ). In their disease models, including sarcoidosis and systemic lupus erythematous, the frequency of CD45RA^-^Foxp3^low^CD4^+^ T cells (non-Tregs) and CD45RA^-^Foxp3^high^ Tregs was reversed [14]. However, in our observations of HNSCC, CD45RA^-^Foxp3^low^CD4^+^ T cells were increased in parallel with CD45RA^-^Foxp3^high^ Tregs in HNSCC patients. We have found that this Treg subset secreted high amount of effector cytokines, but did not have suppressive activity in vitro. We hypothesized that the CD45RA^-^Foxp3^low^CD4^+^ T cells could be a heterogeneous Treg subset in HNSCC. They might be non-Tregs and could differentiate into effector T cells as others have proposed [16]. The increased frequency of this subset might be the result of antigen exposure in tumor microenvironment [29]. Further studies regarding the role of this subset in HNSCC, including the function and differentiation, will be more intriguing in future. Taken together, our data suggest that we should carefully identify distinct Treg subsets rather than the whole population of Tregs in the study of HNSCC, and that CD45RA^-^Foxp3^high^ Tregs might be the potential selective targeting factors in future HNSCC immunotherapy.

HNSCC develop from anatomically defined locations within the upper aerodigestive tract: larynx, hypopharynx, oral cavity, oropharynx, nasopharynx, and nasal cavity. Those tumors arising from different subsites are frequently grouped together in previous research studies [10,27,28], but the various subsites are known to have different etiology and survival rates for the same stage of disease. Hence, it should be necessary to evaluate the variation of Tregs among HNSCC patient subgroups.

The present study showed that there was no significant difference in the frequency of Tregs between OCSCC patients and healthy donors. This is in contrast to the majority of results reported by previous HNSCC studies where Tregs have been found to be increased in the cancer patients [10,22,30,31]. However, not all cancer publications report an elevated trend, with some observing no significant difference in the frequency of Tregs in the peripheral circulation of patients and healthy donors, including one study examining oral SCC [32,33]. It is perhaps not surprising that results between studies are inconsistent, with the use of different markers to identify Tregs and a heterogeneous cancer population. These biological and methodological factors are likely to cause differences in reported Tregs behavior. In spite of the above-described phenomenon, we showed for the first time that the frequency of CD45RA^-^Foxp3^high^ Tregs with suppressive activity in OCSCC patients was higher than in healthy donors. Again, these findings suggest us to identify CD45RA^-^Foxp3^high^ Tregs rather than the whole population of Tregs in the study of HNSCC.

In the study of the association between CD45RA^-^Foxp3^high^ Tregs and tumor sites, the frequency of CD45RA^-^Foxp3^high^ Tregs was similar between patients with HPSCC, NPSCC, OPSCC, and LSCC. These findings were partially in parallel with previous HNSCC investigations which showed that there was no significant association between the frequency of Tregs and tumor sites, including the larynx and oropharynx [24,33]. Although the frequency of CD45RA^-^Foxp3^high^ Tregs did not differ between patients with HPSCC, NPSCC, OPSCC, and LSCC, it was found that HNSCC patients with advanced stage tumors and those that metastasized to the lymph nodes had significantly increased levels of CD45RA^-^Foxp3^high^ Tregs in comparison to patients with early stage tumors and no nodal involvement, respectively; in contrast to previous HNSCC studies which found no differences [10,22-24]. However, recent studies of HNSCC showed that CD127^low/-^ Tregs (including CD4^+^CD25^inter^CD127^low/-^ and CD4^+^CD25^high^ CD127^low/-^ Tregs) or CD4^+^CD25^+^Foxp3^+^ Tregs are associated with advanced stage and nodal involvement [33,34]. This is hypothesized to be due to the different phenotypes used to identify Tregs and the composition of the patient cohorts.

## Conclusions

The present study provides evidence to support the notion of heterogeneous Treg subsets in the peripheral circulation of HNSCC patients. CD45RA^-^Foxp3^high^ Tregs (one distinct Treg subset) significantly increase in the peripheral circulation of HNSCC patient subgroups. Importantly, CD45RA^-^Foxp3^high^ Tregs positively correlate with tumor progression. The present findings provide important information of the future design of immunotherapeutic strategies for HNSCC patients, for example by monoclonal antibodies (anti-PD-1 Ab and anti-CTLA-4 Ab), to reduce the expansion, survival and suppressive function of the Tregs responsible for HNSCC-specific immune suppression – as ever the problem remains effective, specific targeting.

## Competing interests

The authors declare that they have no competing interests.

## Authors’ contributions

WS and WPW conceived and designed the experiments. WS, WJL, and CYW performed the experiments and analyzed the data. WJL performed the statistical analysis. WJL and HZ made substantial contribution to collecting blood samples. WS and WPW wrote the manuscript. All authors have read and approved the final manuscript.

## Supplementary Material

Additional file 1: Figure S1 Relationship between expression levels of CD25 vs. CD45RA and Foxp3 vs. CD45RA in PB CD4+ T cells of HNSCC patients. The degree of CD25 expression in CD45RA + CD25++ Tregs (Fraction 1), CD45RA-CD25+++ Tregs (Fraction 2), and CD45RA-CD25++CD4+ T cells (Fraction 3). (a) are proportional to Foxp3 expression in CD45RA + Foxp3low Tregs (Fraction I), CD45RA-Foxp3high Tregs (Fraction II), and CD45RA-Foxp3low CD4+ T cells (Fraction III), respectively (b). Gating strategy used is illustrated as follows: CD45RA-CD25+ cells with red background fluorescence (x-axis) were defined as CD45RA-CD25+ (CD25low). The CD45RA + CD25++ (CD25inter) gate (Fraction 1) was adjusted to contain CD45RA + T cells that express CD25 more brightly than CD45RA-CD25+ (CD25low). The CD45RA-CD25+++ (CD25high) gate (Fraction 2) was adjusted to contain CD45RAT cells exceeding the level of CD25 expression on CD45RA + CD25++ (CD25inter) cells. The CD45RA-CD25++ (CD25inter) gate (Fraction 3) was adjusted to contain CD45RAT cells with the same level of CD25 expression as CD45RA + CD25++ (CD25inter) cells. Click here for file

Additional file 2: Figure S2 Cytokine production by responder T cells. The histograms represent the cytokine expression profiles of responder cells co-cultured with CD45RA + CD25++, CD45RA-CD25+++, or Tregs CD45RA-CD25++CD4+ T cells (P > 0.05). Data are representative of 9 separate experiments. Statistical comparisons were performed using the Student’s t-test. Click here for file
