# Supplementary material for: CD45RA-Foxp3high but not CD45RA+Foxp3low suppressive T regulatory cells increased in the peripheral circulation of patients with head and neck squamous cell carcinoma and correlated with tumor progression
Source: J Exp Clin Cancer Res. 2014 Apr 25;33(1):35. doi: 10.1186/1756-9966-33-35 (PMC4022051; doi:10.1186/1756-9966-33-35)
Supplement: Additional file 1: Figure S1 — Relationship between expression levels of CD25 vs. CD45RA and Foxp3 vs. CD45RA in PB CD4+ T cells of HNSCC patients. The degree of CD25 expression in CD45RA + CD25++ Tregs (Fraction 1), CD45RA-CD25+++ Tregs (Fraction 2), and CD45RA-CD25++CD4+ T cells (Fraction 3). (a) are proportional to Foxp3 expression in CD45RA + Foxp3low Tregs (Fraction I), CD45RA-Foxp3high Tregs (Fraction II), and CD45RA-Foxp3low CD4+ T cells (Fraction III), respectively (b). Gating strategy used is illustrated as follows: CD45RA-CD25+ cells with red background fluorescence (x-axis) were defined as CD45RA-CD25+ (CD25low). The CD45RA + CD25++ (CD25inter) gate (Fraction 1) was adjusted to contain CD45RA + T cells that express CD25 more brightly than CD45RA-CD25+ (CD25low). The CD45RA-CD25+++ (CD25high) gate (Fraction 2) was adjusted to contain CD45RAT cells exceeding the level of CD25 expression on CD45RA + CD25++ (CD25inter) cells. The CD45RA-CD25++ (CD25inter) gate (Fraction 3) was adjusted to contain CD45RAT cells with the same level of CD25 expression as CD45RA + CD25++ (CD25inter) cells. [file 1756-9966-33-35-S1.pdf]

Supplementary Figure 1 The correlation of expression levels of CD25 vs. CD45RA and FoxP3 vs. CD45RA in HNSCC patients

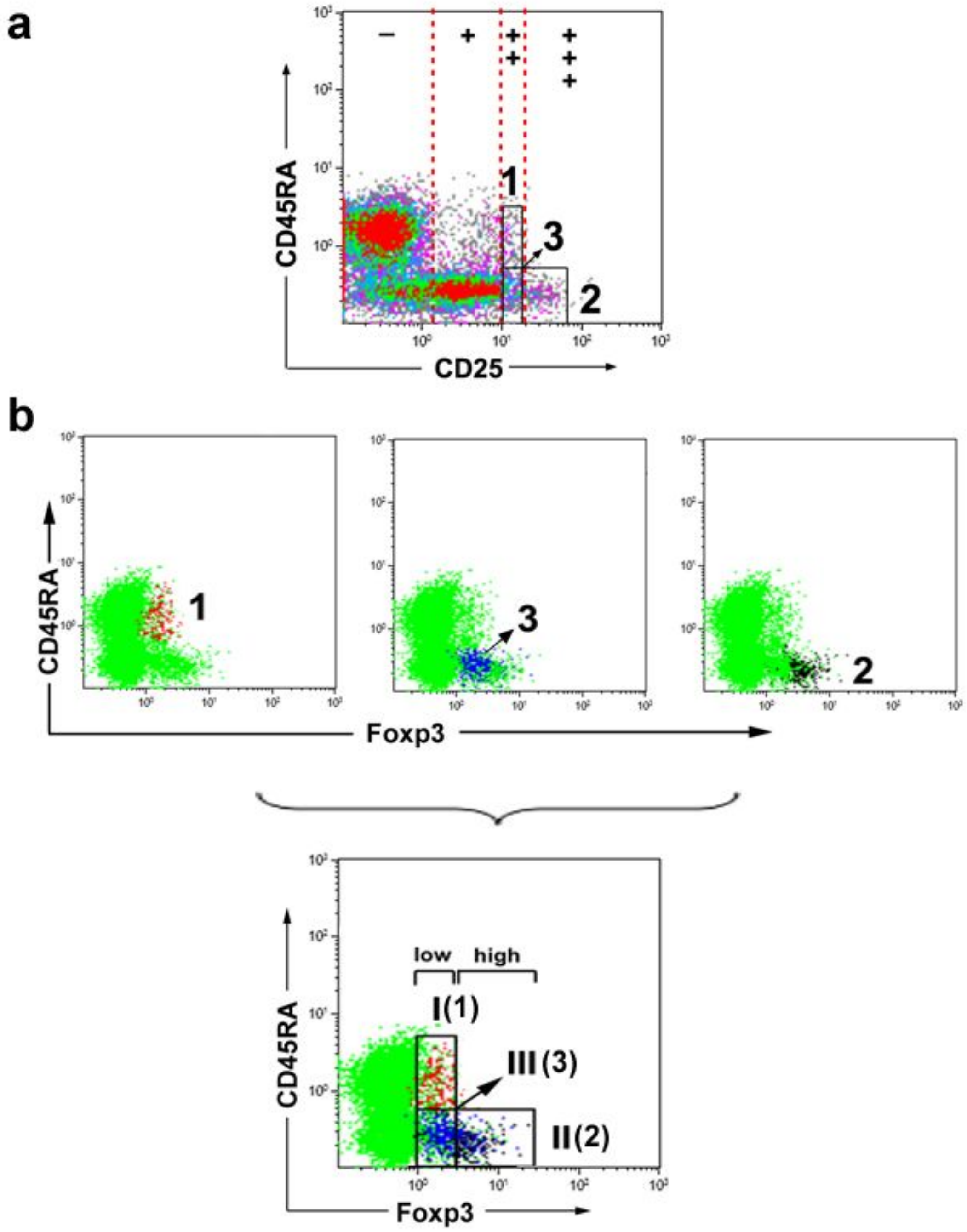

Supplementary Figure 1. Relationship between expression levels of CD25 vs. CD45RA and Foxp3 vs. CD45RA in PB CD4^{+} T cells of HNSCC patients. The

degree of CD25 expression in $CD45RA^{+}CD25^{++}$ Tregs (Fraction 1), $CD45RA^{-}CD25^{+++}$ Tregs (Fraction 2), and $CD45RA^{-}CD25^{++}CD4^{+}$ T cells (Fraction 3) **(a)** are proportional to Foxp3 expression in $CD45RA^{+}Foxp3^{low}$ Tregs (Fraction I), $CD45RA^{-}Foxp3^{high}$ Tregs (Fraction II), and $CD45RA^{-}Foxp3^{low}$ $CD4^{+}$ T cells (Fraction III), respectively **(b)**. Gating strategy used is illustrated as follows: $CD45RA^{-}CD25^{+}$ cells with red background fluorescence (x-axis) were defined as $CD45RA^{-}CD25^{+}$ ($CD25^{low}$). The $CD45RA^{+}CD25^{++}$ ($CD25^{inter}$) gate (Fraction 1) was adjusted to contain $CD45RA^{+}$ T cells that express CD25 more brightly than $CD45RA^{-}CD25^{+}$ ($CD25^{low}$). The $CD45RA^{-}CD25^{+++}$ ($CD25^{high}$) gate (Fraction 2) was adjusted to contain $CD45RA^{-}$ T cells exceeding the level of CD25 expression on $CD45RA^{+}CD25^{++}$ ($CD25^{inter}$) cells. The $CD45RA^{-}CD25^{++}$ ($CD25^{inter}$) gate (Fraction 3) was adjusted to contain $CD45RA^{-}$ T cells with the same level of CD25 expression as $CD45RA^{+}CD25^{++}$ ($CD25^{inter}$) cells.
