# Supplementary material for: CD45RA-Foxp3high but not CD45RA+Foxp3low suppressive T regulatory cells increased in the peripheral circulation of patients with head and neck squamous cell carcinoma and correlated with tumor progression
Source: J Exp Clin Cancer Res. 2014 Apr 25;33(1):35. doi: 10.1186/1756-9966-33-35 (PMC4022051; doi:10.1186/1756-9966-33-35)
Supplement: Additional file 2: Figure S2 — Cytokine production by responder T cells. The histograms represent the cytokine expression profiles of responder cells co-cultured with CD45RA + CD25++, CD45RA-CD25+++, or Tregs CD45RA-CD25++CD4+ T cells (P > 0.05). Data are representative of 9 separate experiments. Statistical comparisons were performed using the Student’s t-test. [file 1756-9966-33-35-S2.pdf]

Supplementary Figure 2 Cytokine expression profiles of responder T cells

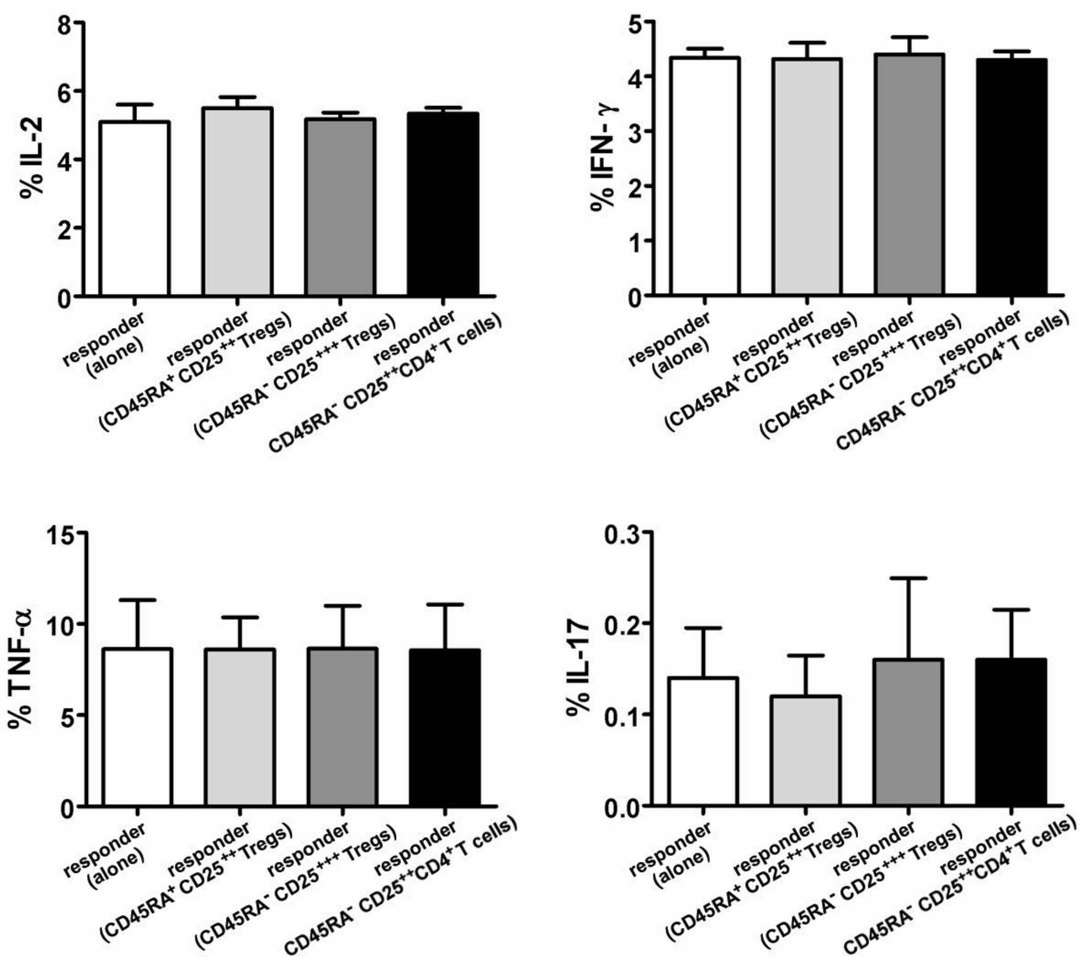

Supplementary Figure 2. Cytokine production by responder T cells. The histograms represent the cytokine expression profiles of responder cells co-cultured with CD45RA^{+}CD25^{++}, CD45RA^{-}CD25^{+++}, or Tregs CD45RA^{-}CD25^{++}CD4^{+} T cells ($P > 0.05$). Data are representative of 9 separate experiments. Statistical comparisons were performed using the Student’s t-test.
